# Development and validation of a radiopathomic model for predicting pathologic complete response to neoadjuvant chemotherapy in breast cancer patients

**DOI:** 10.1186/s12885-023-10817-2

**Published:** 2023-05-12

**Authors:** Jieqiu Zhang, Qi Wu, Wei Yin, Lu Yang, Bo Xiao, Jianmei Wang, Xiaopeng Yao

**Affiliations:** 1 School of Public Health, Southwest Medical University, Luzhou, China; 2 Department of Pathology, The Affiliated Hospital of Southwest Medical University, Luzhou, China; 3 Department of Radiology, The Affiliated Hospital of Southwest Medical University, Luzhou, China; 4 Nuclear Medicine and Molecular Imaging Key Laboratory of Sichuan Province, Luzhou, China; 5 School of Medical Information and Engineering, Southwest Medical University, Luzhou, China; 6 Central Nervous System Drug Key Laboratory of Sichuan Province, Southwest Medical University, Luzhou, China

**Keywords:** Breast cancer, Radiomics, Pathomics, Radiopathomics, CECT

## Abstract

**Background:**

Neoadjuvant chemotherapy (NAC) has become the standard therapeutic option for early high-risk and locally advanced breast cancer. However, response rates to NAC vary between patients, causing delays in treatment and affecting the prognosis for patients who do not sensitive to NAC.

**Materials and methods:**

In total, 211 breast cancer patients who completed NAC (training set: 155, validation set: 56) were retrospectively enrolled. we developed a deep learning radiopathomics model(DLRPM) by Support Vector Machine (SVM) method based on clinicopathological features, radiomics features, and pathomics features. Furthermore, we comprehensively validated the DLRPM and compared it with three single-scale signatures.

**Results:**

DLRPM had favourable performance for the prediction of pathological complete response (pCR) in the training set (AUC 0.933[95% CI 0.895–0.971]), and in the validation set (AUC 0.927 [95% CI 0.858–0.996]). In the validation set, DLRPM also significantly outperformed the radiomics signature (AUC 0.821[0.700–0.942]), pathomics signature (AUC 0.766[0.629–0.903]), and deep learning pathomics signature (AUC 0.804[0.683–0.925]) (all *p* < 0.05). The calibration curves and decision curve analysis also indicated the clinical effectiveness of the DLRPM.

**Conclusions:**

DLRPM can help clinicians accurately predict the efficacy of NAC before treatment, highlighting the potential of artificial intelligence to improve the personalized treatment of breast cancer patients.

**Supplementary Information:**

The online version contains supplementary material available at 10.1186/s12885-023-10817-2.

## Introduction

Breast cancer is the most common cancer among women in the world, and it related incidence rate is continuously rising [1, 2]. Neoadjuvant chemotherapy (NAC) has become the standard therapeutic option for early high-risk and locally advanced breast cancer [3]. When breast cancer patients have a pathological complete response (pCR) to NAC, it can help patients lower the stage and shrink the tumor to receive more conservative treatment, and its event free survival (EFS) and overall survival (OS) are significantly improved [4, 5]. However, because of the heterogeneity and complexity of tumors, not all patients benefit from NAC. For patients who are not sensitive to treatment, although disease progression rarely occurs during NAC [6], the long-term treatment process will still have side effects [7, 8], which may also lead to missing the best time to change the treatment plan. Currently, there is an urgent requirement for accurate prediction of the response before the NAC, which is critical for breast cancer patients who are destined to have no response.

Radiomics could predict effectively pCR in patients with breast cancer. In the process of NAC, some studies used the images of different treatment nodes to fuse each other to predict pCR [9, 10], but the image data required for model construction was obtained after NAC, and the clinical practicability of the model was poor. Some scholars have made optimization on this basis [11, 12], it could benefit patients in some extent, but the radiomics features only provide tumor information from a macroscopic perspective in vitro, it cannot completely reflect information about pCR. As another source of medical images, histopathology combined with machine learning can help in risk stratification, prognosis prediction and adjuvant chemotherapy efficacy prediction [13–16]. Pathomics differ from radiomics in that they provide microstructural information about the tumor microenvironment, which can complement tumor heterogeneity and enhance the predictive power of existing models. So we hypothesized that a multi-scale model integrating the features of radiomics and pathomics could efficiently predict pCR.

In this study, we aimed to develop and validate a deep learning radiopathomics model(DLRPM) for the prediction of pCR to NAC in patients with breast cancer using Contrast-Enhanced Computed Tomography (CECT) images and whole slide images (WSIs). This proposed DLRPM can be used for early adjustment therapy in non-PCR patients to improve pCR rates and avoid toxic side effects. This might provide clinicians with treatment strategies to improve the effectiveness of individual therapy.

## Materials and methods

### Patients

This retrospective study was approved by the Institutional Review Board of the Southwestern Medical University Hospital (No. KY2022216), and the requirement for written informed consent was waived. This study collected 1532 patients with breast cancer who underwent a CECT examination between January 2020 and March 2022 from the Picture Archiving and Communications System (PACS).

The inclusion criteria were as follows: (a) Pathological biopsy confirmed non-specific invasive breast cancer with no distant metastasis; (b) The patient has undergone 6–8 cycles of NAC; (c) surgery was performed after NAC; (d) The available clinical data. A total of 245 patients fulfilling the inclusion criteria were enrolled.

Exclusion criteria were as follows: (a)No histopathological evaluation results. (b) Lack of images in venous phase or poor image quality. (c)Synchronous tumors or history of other malignancy.

**Fig. 1:**
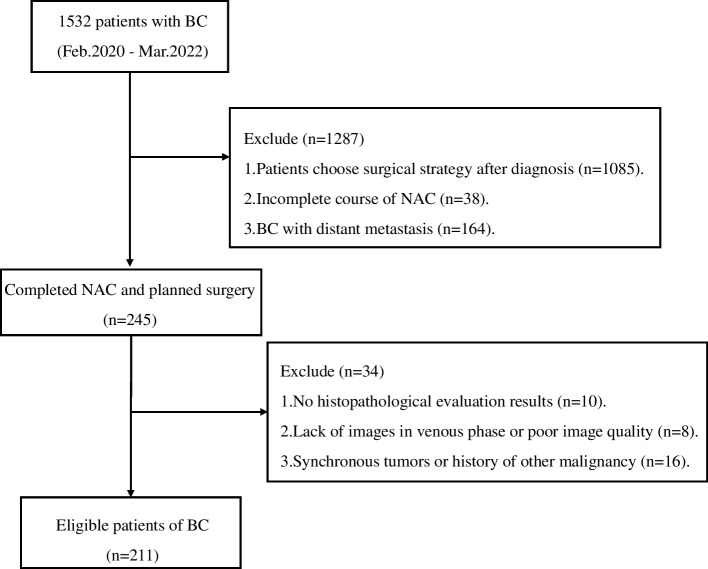
Flow diagram of patient cohort selection

A total of 211 breast cancer patients with non-specific invasive were enrolled in the study from February 2020 and March 2022, we divided patients into training set and independent validation set in chronological order. Patients who performed their first NAC treatment before September 2021 were used as the training set, the remaining patients were used as the validation set, the ratio of training set to validation set was about 7:3. A flowchart of the patients’ collection is shown in Fig. 1.

### Workflow of study

**Fig. 2:**
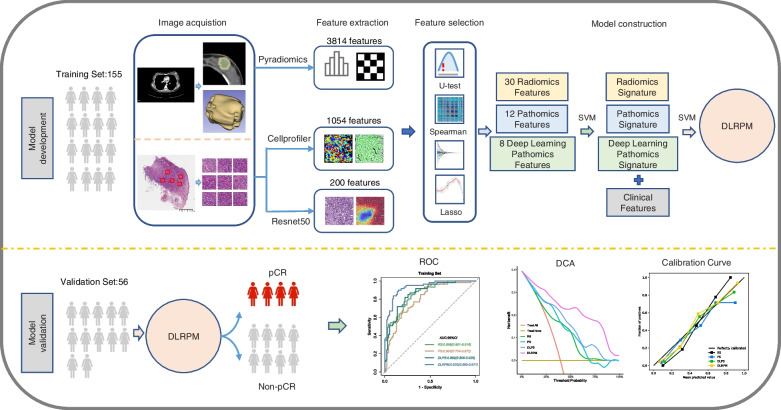
Workflow of Study. The images were preprocessed for feature extraction. After feature evaluation and model construction, four sets of features [radiomic signature (RS), pathologic signature (PS), deep learning pathologic signature (DLPS) and clinical features] were generated and further used to construct DLRPM. The performance of DLRPM in predicting pCR before NAC was validated in validation set

The workflow of this study is shown in Fig. 2, including (1) image acquisition, (2) feature extraction, (3) feature selection, (4) model construction, and (5) model validation.

### The CECT images and WSIs acquisition

All patients received contrast-enhanced CT chest examination (Netherlands, Philips Medical Systems) before NAC treatment. The scanning procedure was as follows: The contrast agent (iodohexol, 320 mg/mL) was injected into the median cubital vein with a double-barrel high-pressure syringe (dose 1.0 mL/kg, flow rate 3.0 mL/s). The CT value of blood vessels at the level of the aortic arch was monitored after injection of contrast agent. The Enhanced CT scans are automatically triggered when the CT value reaches around 250 HU. And venous phase scans were performed after a delay of 30s.

The pathologists collected all biopsy samples of breast cancer patients using crude needle puncture before NAC. Firstly, biopsy tissue was soaked in 10% formalin for 4 h and buried in immunohistochemical paraffin wax. Subsequently, the biopsied tissue was severed at 4-μm intervals and stained with hematoxylin and eosin (H&E) for pathological evaluation. A pathologist with 8 years of experience in pathological diagnosis scanned all H&E-stained histopathological slides using a digital slide scanner (KFBio KF-PRO-020) at 10 × magnification to obtain WSIs of the breast cancer patients, and images were digitized as kbf. format files, which were managed with the KF-Viewer software (version 1.7.0.23).

### Pathological complete response assessment

**Fig. 3:**
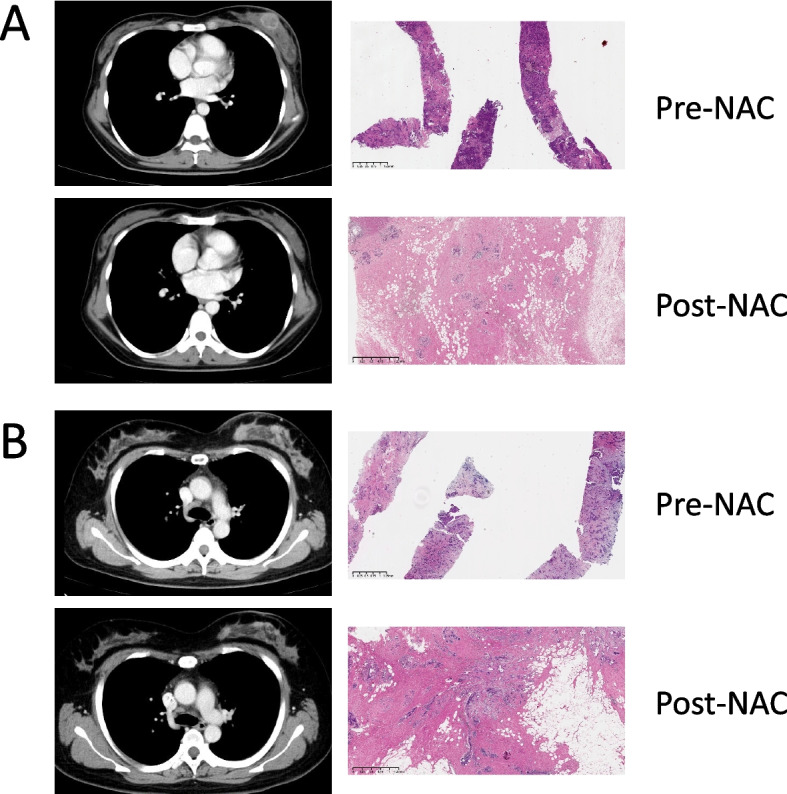
CECT and histology images from complete responder (**A**) and partial responder (**B**) before NAC and after 8 courses of NAC. In the CECT image, it is seen that the tumor in the complete responder have completely dissipated in the post-NAC image, and stromal tissue with no visible tumor cells was presented in the pathological images. But CECT and histology images from and partial responder shows residual tumor cells but reduced compared to baseline

In accordance with the National Comprehensive Cancer Network (NCCN) guideline [17], all patients received six or eight cycles of NAC. The NAC regimens were based on taxane or taxane and anthracycline; all human epidermal growth factor receptor 2 (HER2) positive patients also received trastuzumab. At the end of treatment, we performed an initial imaging assessment of the efficacy of NAC according to the Response Evaluation Criteria in Solid Tumors (RECIST) 1.1 [18]. Subsequently, the final pCR status of each patient was determined by the pathological findings after surgery (Fig. 3). pCR was defined as the complete absence of invasive tumor cells in the breast and axillary lymph nodes, regardless of the presence of residual ductal carcinoma in situ (ypT0/isypN0) (Fig. 3A).

### Radiomics feature extraction

The volume of interest (VOI) segmentation was performed using 3D-Slicer software. All manual segmentation of the CECT images were performed by 2 practicing experienced radiologists. Both radiologists were blinded to the patient's clinical data when they evaluated the CECT images. Firstly, the VOIs covering the whole tumor (VOI 1) were segmented. After manual tumor segmentation, we automatically segment the peritumoral regions (VOI 2) (Figure S1). The regions (2-mm radius) surrounding the tumor were defined as the peritumoral regions. If the peritumor regions were beyond the parenchyma of the breast after the spread, the portion beyond the parenchyma was removed manually.

According to the instructions of the Image Biomarker Standardization Initiative [19], radiomics features were extracted from the VOI 1 and VOI 2 using PyRadiomics, before feature extraction, PyRadiomics was also used for image preprocessing. A total of 3814 radiomics features were extracted from two VOIs per patient. These features included First Order, Shape-based (2D and 3D), Gray Level Cooccurence Matrix (GLCM), Gray Level Run Length Matrix (GLRLM), Gray Level Size Zone Matrix (GLSZM), Gray Level Dependece Matrix (GLDM); and Filters included Laplacian of Gaussian (LoG), Wavelet, Square, Square Root, Logarithm, Exponential, Gradient, Local Binary Pattern (LBP) 3D. To make features reproducible, an interclass correlation coefficient (ICC) higher than 0.75 was considered credible [20].

### Pathomics feature extraction

In KF Viewer, WSIs were magnified 10 × , the pathologist (W.Q) selected the sample area containing nuclear pleomorphism, mitosis, carcinoma infiltration, cancer invasion, tumor cell differentiation and pathological grading, and obtained five typical non-overlapping screenshots with a field of vision of 1534 × 832 pixels, and then confirmed by the other pathologist (J.M).They had 3 years and 8 years of experience in breast cancer pathological diagnosis respectively. We saved the selected screenshots as format files (. jpg, 300dpi). If the two pathologists have different opinions, they will consult the third pathologist to make a decision. All screenshots were cut into small frame tiles (512 × 512 pixels) by sampling without overlap for subsequent analysis (Figure S2).

We used CellProfiler (version 4.0.7) [21], an open-source image analysis software developed by Broad Institute (Cambridge, Massachusetts), to extract quantitative pathomics features of selected pathological screenshots. Based on the “Unmix Colors” module to separate H&E-stained images and convert them into haematoxylin-stained and eosin-stained greyscale images, The H&E-stained images were also converted to greyscale images using the “ColorToGray” module (Figure S3). We measured images twice, in the first measurement, we obtained 136 original features, which summarize the three types of images in general. For the second measurement, we made a careful exploration of hematoxylin images. First, we identified the primary and secondary objects, and then measured them. After measurement, we took their mean, median and standard deviation as our research characteristics and 1054 pathomics features were obtained, the extracted features were aggregated by mean of the values for every 10 tiles in each WSI. Detailed method of feature extraction in Figure S4.

Resnet50 was employed to extract deep learning features of pathomics. Before extracting features, All the small tiles went through color normalization with the Vahadane method based on staintools (Figure S2), which was an open-source package based on python for stain normalization and augmentation. The input area was 512 × 512 pixels, and the transfer learning took the pretrained weights of Resnet50 on the ImageNet dataset as the initial weights of the model, The model was fine-tuned using data from our data. Resnet50 was adjusted from the original multi-classification task to a binary classification task, we extracted the deep learning features from the last layer of resnet50, and the principal component analysis (PCA) algorithm further compressed the deep learning features, the extracted features were aggregated by mean of the values for every 10 tiles in each WSI. We obtained a total of 200 deep learning features.

### Feature selection and signature construction

The radiomics, pathomics features and pathomics deep learning features can reveal tumour information from macroenvironment and microenvironment perspectives, repectively. However, these features were high-dimensional data, which had an adverse impact on predicting the pCR to NAC. Therefore, we should obtain the features which were most closely related to pCR in the training set. Firstly, all variables were normalized, and a U-test was performed on each feature as a preliminary selection to remove redundant features. To sufficiently extract discriminative features in this process, the threshold of *p* value was determined with 0.05. Subsequently, considering the dependence between features, we perform correlation analysis on the features, if the correlation coefficient between the two features was greater than 0.9, one of them was excluded. Then, the least absolute shrinkage and selection operator (LASSO) algorithm was utilized to select the extracted features [22], and tenfold cross-validation was used to select the value of Lambda to determine the optimal features.

Based on the above three types of optimal features, we constructed three distinct single-scale prediction models by Support Vector Machine (SVM) method [23], The best regularization parameter C and Gamma (γ) for Gaussian Radial Basis Function (RBF) kernel were determined by fivefold cross validation and grid search. Then, each model prediction value was used to construct signatures, named radiomics signature (RS), pathomics signature (PS), and deep learning pathomics signature (DLPS), respectively.

### DLRPM development and validation

Independent clinical predictor and three single-scale signatures were used to construct the DLRPM for the integrated prediction of pCR in breast cancer patients by a similar non-linear SVM method. We used the following methods to comprehensively evaluate the model. Receiver operating characteristic (ROC) curve analysis, sensitivity, specificity, positive predictive value (PPV), and negative predictive value (NPV) were employed to evaluate the discrimination performance [24]. The calibration of models were assessed using the calibration curve and the Hosmer–Lemeshow test was used to assess the goodness-of-fit of the models.The Decision curve analysis (DCA) of all models was performed to quantify the net benefit of patients under different threshold probabilities in the sets to assess the clinical value of the predictive models in our study [25].

In addition, the net reclassification improvement (NRI) test and integrated discrimination improvement (IDI) test were calculated to compare the performance of DLRPM and single-scale signatures.

### Statistical analysis

All statistical analysis was performed using R studio (version 4.1.1; R Studio, http://www.R-project.org) and Jupyter Notebook (version 6.4.11). Differences of categorical variables were calculated with the chi-square test or Fisher’s exact test. The differences of continuous variables were analyzed using independent t-test or Mann–Whitney U test. All tests were two-sided, and two-tailed *p* < 0.05 were considered statistically significant.

## Results

### Demographic and clinicopathological characteristics

The baseline characteristics of all patients are summarized in Table 1. A total of 211 patients with breast cancer were enrolled in this study, patients with pCR accounted for 39.35% (61/155) and 37.50% (21/56) of the training and validation sets, respectively, the findings on HER2 was significantly correlated with the pCR status in both the training and validation set (*p* < 0.05). In addition, there were no statistically significant differences between the two sets (*p* > 0.05) (Table S1).

**Table 1:** Clinical characteristics of patients

Characteristics	Training set(*n* = 155)		*p*	Validation set(*n* = 56)		*p*
	Non-pCR(*n* = 94)	pCR(*n* = 61)		Non-pCR(*n* = 35)	pCR(*n* = 21)	
Age	49.5(45.2,55.8)	49.0(46.0,54.0)	0.437	52.3 ± 9.63	50.3 ± 9.26	0.623
T stage (%)			0.149			0.420
T1	4 (4.26)	3 (4.92)		3 (8.57)	0 (0.00)	
T2	64 (68.1)	47 (77.0)		23 (65.7)	18 (85.7)	
T3	10 (10.6)	8 (13.1)		5 (14.3)	2 (9.52)	
T4	16 (17.0)	3 (4.92)		4 (11.4)	1 (4.76)	
N stage (%)			0.076			0.946
N0	28 (29.8)	13 (21.3)		9 (25.7)	5 (23.8)	
N1	50 (53.2)	44 (72.1)		17 (48.6)	12 (57.1)	
N2	13 (13.8)	4 (6.56)		6 (17.1)	2 (9.52)	
N3	3 (3.19)	0 (0.00)		3 (8.57)	2 (9.52)	
ER status (%)			0.03			0.128
Negative	39 (41.5)	37 (60.7)		13 (37.1)	13 (61.9)	
Positive	55 (58.5)	24 (39.3)		22 (62.9)	8 (38.1)	
PR status (%)			0.047			0.448
Negative	30 (31.9)	30 (49.2)		15 (42.9)	12 (57.1)	
Positive	64 (68.1)	31 (50.8)		20 (57.1)	9 (42.9)	
HER2 status (%)			< 0.001			0.001
Negative	69 (73.4)	22 (36.1)		25 (71.4)	5 (23.8)	
Positive	25 (26.6)	39 (63.9)		10 (28.6)	16 (76.2)	
Ki-67 status (%)			0.238			1.000
< 30%	26 (27.7)	11 (18.0)		8 (22.9)	5 (23.8)	
≥ 30%	68 (72.3)	50 (82.0)		27 (77.1)	16 (76.2)	

Data in parentheses are percentages; *p* values were derived from the univariate analysis between each of characteristic and pCR Status. *HER2* Human epidermal growth factor receptor 2, *ER* Estrogen receptor, *PR* Progesterone receptor

### Radio-pathomics feature selection and signature construction

The inter-observer reproducibility of the feature extraction was excellent, with inter-observer ICCs ranging from 0.758 to 0.953 for CECT. U-test and Spearman correlation coefficient analysis was performed exclude redundant features, which resulted in 154 radiomic features, 21 pathomics Feature and 8 deep learning Pathomics Feature per patient.

**Fig. 4:**
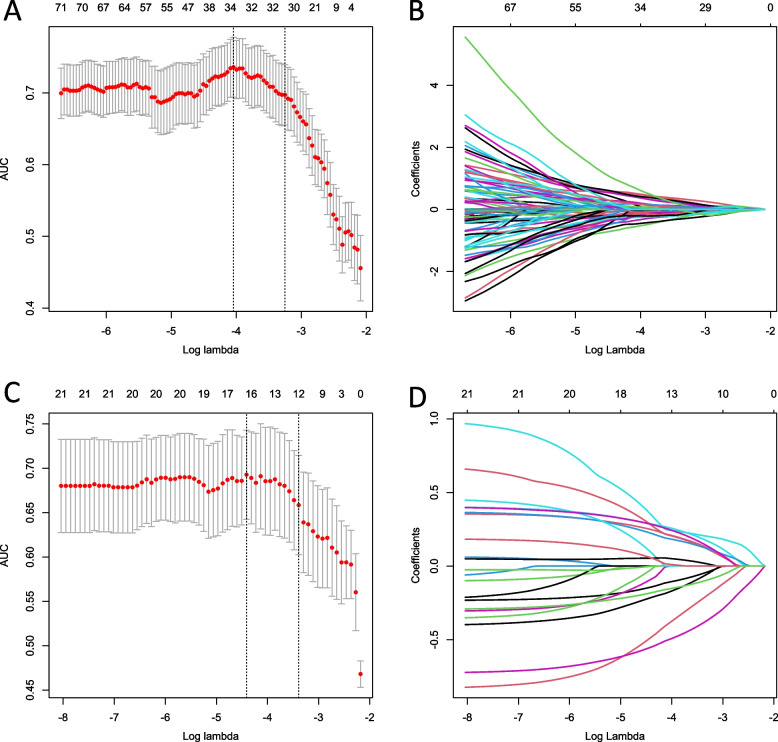
Feature selection process. Radiomics features (**A**, **B**) and pathomics features (**C**, **D**) were selected by the LASSO model with tuning parameter (λ) using fivefold cross-validation via minimum and 1se criteria

Then, LASSO was adopted to deeply select the pre-existing features. For LASSO, the subset of the best features depended on the choice of lambda value, and we used fivefold cross validation to find the best lambda value. To further simplify the model, step-forward feature selection was then conducted to reduce the optimal features, we pick the lambda value with one standard error to select features, which resulted in 30 radiomic features and 12 pathomics Feature, for deep learning pathomics Feature, Lasso did not further reduce features (Fig. 4) (Table S2, 3).

**Fig. 5:**
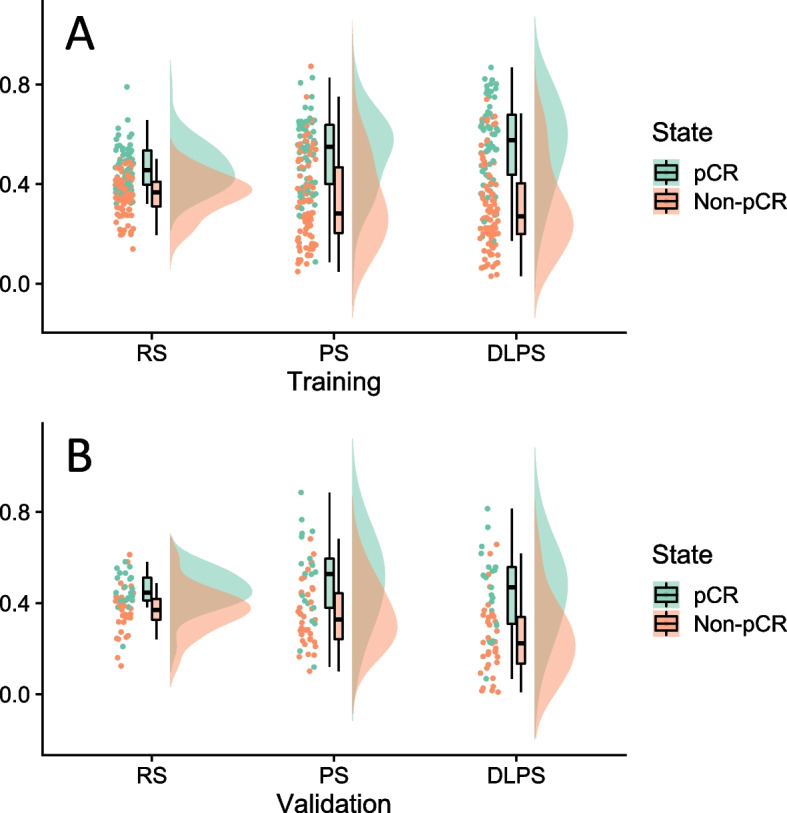
The raincloud plot visualizes prediction probability of RS, PS and DLPS, it shows the sample distribution locations and interval sample densities for the training (**A**)and validation set(**B**) of signatures

We constructed three predictive signatures by non-linear SVM method respectively. In training set, the grid search with fivefold cross validation found the optimal parameters of the three models, RS (C = 4.67, gamma = 0.0015), PS (C = 1, gamma = 0.0028) and DLPS (C = 2.15, gamma = 0.031). Raincloud plot (Fig. 5) visualized the different distributions of the samples in training and validation sets, it indicated Three single-scale signatures already had a certain discriminant ability (*p* < 0·05, Table 2).

**Table 2:** Predicted probability of signatures and in training and validation set

Training Set	Non-pCR(*n* = 94)	pCR(*n* = 61)	*p*
RS	0.31(0.20,0.41)	0.46(0.40,0.71)	< 0.001
PS	0.28(0.19,0.44)	0.55(0.38,0.63)	< 0.001
DLPS	0.21(0.15,0.37)	0.60(0.44,0.66)	< 0.001
Validation Set	Non-pCR(*n* = 35)	pCR(*n* = 21)	*p*
RS	0.34 ± 0.18	0.54 ± 0.16	< 0.001
PS	0.34 ± 0.14	0.51 ± 0.20	0.002
DLPS	0.19(0.10,0.35)	0.44(0.29,0.56)	< 0.001

Skewed distributed variable values: median (interquartile range), Normally distributed variable values: mean ± standard deviation

### DLRPM development and validation

**Fig. 6:**
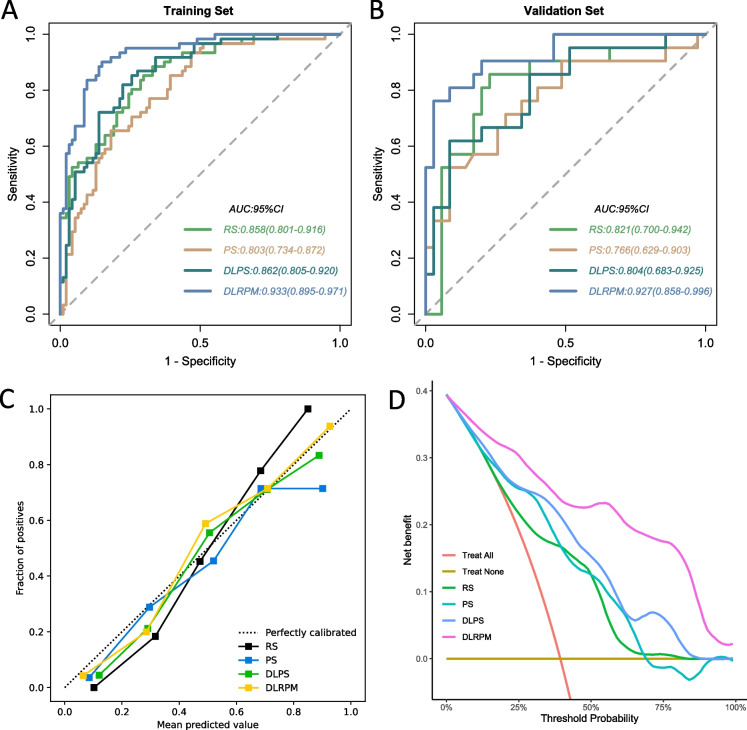
ROC analysis of predict models for predicting pCR in the training set (**A**) and validation set (**B**), respectively. **C** Calibration curves of models in training set on discriminating Non-pCR versus pCR. **D** Decision curve analysis in training set using RS, PS, DLPS and DLRPM

By integrating HER2, RS, PS and DLPS in the training set, we developed the DLRPM comprehensive prediction model by using the nonlinear support vector machine method. The same method was used to find the optimal parameter of DLRPM (C = 5, gamma = 0.01). ROC curves were used to assess discrimination performance, DLRPM accurately predicted pCR in Training set (AUC 0·933[95% CI 0.895–0.971]) and validation set (0.927 [95% CI 0.858–0.996]). The sensitivity of DLRPM was markedly high in validation set (94.28%), whereas the specificity remained moderate (76.19%). The NPV of DLRPM exceeded 90% in validation set, whereas the PPV was around 71.25% (Table 3, Fig. 6A).

**Table 3:** Discrimination performance of predict models for predicting pCR status in breast cancer patients

Training Set	AUC (95%CI)	SEN (%)	SPE (%)	ACC (%)	PPV (%)	NPV (%)
HER2	0.686(0.611–0.762)	63.93	73.04	69.67	60.93	75.82
RS	0.858(0.801–0.916)	88.52	67.02	75.48	63.52	90.00
PS	0.803(0.734–0.872)	65.57	81.91	75.48	70.17	78.57
DLPS	0.862(0.805–0.920)	85.24	74.46	78.70	68.42	88.60
DLRPM	0.933(0.895–0.971)	90.16	85.10	87.09	79.71	93.02
Validation Set	AUC (95%CI)	SEN (%)	SPE (%)	ACC (%)	PPV (%)	NPV (%)
HER2	0.738(0.617–0.858)	76.19	71.42	73.21	61.53	83.33
RS	0.821(0.700–0.942)	85.71	77.14	80.35	69.23	90.00
PS	0.766(0.629–0.903)	52.38	91.42	76.78	78.57	76.19
DLPS	0.804(0.683–0.925)	61.90	91.42	80.35	81.25	80.00
DLRPM	0.927(0.858–0.996)	94.28	76.19	87.50	71.25	92.25

*AUC* Area under the receiver operating curve, *CI* Confidence interval, *SEN* sensitivity, *SPE* Specificity, *ACC* Accuracy, *PPV* Positive predictive value, *NPV* Negative predictive value

In validation set, RS and DLPS yielded marginally AUC values of 0.821(0.700–0.942) and 0.804(0.683–0.925) (Fig. 6B), whereas PS had a lower AUC value of 0.766(0.629–0.903). the DeLong test, NRI and IDI showed that performance of the three single scale models had no significant difference(all *p* > 0.05).

Compared with single-scale prediction models, DLRPM showed superior to evaluate the discrimination performance. The improvements in discriminative ability were confirmed by the NRI tests (all *p* < 0·05) and IDI (all *p* < 0·05). Calibration plots demonstrated good agreement between all model prediction and the actual observation for detecting pCR (Fig. 6C). The Hosmer–Lemeshow test showed non-significant statistics in both groups (*p* > 0.05). The DCA plots showed that the DLRPM provided better net benefit compared with single-scale prediction models, it indicated DLRPM had a better clinical benefit (Fig. 6D).

## Discussion

In this study, we developed a multi-scale integrated model for prediction of pCR to NAC in breast cancer patients before treatment based on SVM algorithm, combining CECT images with WISs. In the independent validation set, DLRPM had a better performance in terms of discriminative ability, calibration, and clinical utility relative to other single-scale models. In clinical practice, patients who predicted for pCR using DLRPM should be given aggressive NAC therapy, and intensive follow-up strategies were used to improve survival and quality of life. DLRPM assisted clinicians predict accurately the efficacy of NAC treatment before it is administered, which is critical for developing patient treatment plans and optimizing overall patient management.

Predictive biomarkers of response to NAC for breast cancer patients have been the focus topic of research [26], but not all biomarkers were applicable to clinical practice. Previous studies have explored the predictive effectiveness of genetic biomarkers, but they have not been applied in clinical practice because they are costly and and not widespread [27, 28]. Secondly, we should consider the predictive power of the model. Clinicopathological factors were currently used to estimate the potential benefit of NAC, however, satisfactory performance cannot be achieved based on clinical characteristics alone [29–31]. Full digitalization of the stained tissue sections has become feasible because of advances in slide scanning technology and reductions in the cost of digital storage. In our study, DLRPM not only had good predictive power (AUCs > 0.9), but DLRPM had a stable source of modeling and validation data in clinical practice, providing assurance for subsequent large-scale studies.

Previous studies had demonstrated that radiomic biomarker can predict pCR to NAC in breast cancer patients. Many studies applied pre-treatment radiomic features combined with clinicopathological factors for efficacy prediction, but their predictive efficacy was unstable [32–34]; others predicted pCR using pretreatment and post-treatment US images, and their model had better predictive efficacy in the external validation set [9], but promising results were mainly attributed to post-treatment US images, which provided direct information on tumor regression, and the model was unable to provide early estimates of treatment response to guide the implementation of NAC because the images required for the construction of model were obtained late and clinical utility was poor. In contrast, the US images from the second and fourth courses of treatment were matched with US images before treatment, respectively, and This staged prediction pipeline benefited patients to a certain extent [11], and treatment regimens were adjusted based on the prediction results. However, for patients with breast cancer, accurate prediction of neoadjuvant chemotherapy efficacy before treatment can help maximise patient’s benefit.

In the pathological examination, the pathologist used light microscope to determine the benign and malignant tumor, the growth mode and differentiation degree of tumor cells under the light microscope. Because of the limitation of microscope magnification, pathologists were not able to describe the microscopic information of each slide in detail. In recent years,with the application of cell analysis software and the development of deep learning algorithms [14, 15], several scholars researchers have extracted image features from digital pathology slices for quantitative analysis. Breast cancer was a highly heterogeneous tumor, and tumor microenvironment will change when tumor responds to NAC [35]. This subtle change was not detectable by the naked eye. Pathomics can capture the microstructure of tumors and provide the characteristics of cells and microenvironment in tumor lesions. Previous studies had shown pathomics features were used to predict the efficacy and prognosis of adjuvant chemotherapy for gastric cancer [16], and have achieved good performance. In our study, pathomics features also showed a stable predictive ability in predicting the pCR to NAC.

DLRPM integrates macroscopic radiomics and microscopic pathomics features for integrated prediction of pCR to NAC in breast cancer patients. In the radiomics workflow, we added a 2 mm peritumoral region for feature extraction, and the great predictive potential of the peritumoral region features had been demonstrated in the Ning’s study [36]. In our study, the peritumoral region features accounted for 50% of the total radiomics features selected by LASSO and contributed significantly to the predictive power. In the pathomics workflow, we learned from the experience of the current study in CellProfile extraction and performed two extractions of our pathology images. In our analysis, we found that the predictive power of the two types of features can complement each other. DLRPM has high sensitivity and NPV in the independent validation set, indicating that the model can reliably identify individuals without pathological complete response. This study is consistent with the findings of Li’s study [37]. and they could avoid subsequent ineffective treatments, breast cancer patients who were destined not to respond will benefit from the predicted results of DLRPM.

Although our research is innovative, there were also several limitations in our current study. Firstly, this study is a retrospective study, and all of breast cancer patients were obtained from a single medical institution. Considering the limited number of study samples, we will further obtain a large number of sample data from multiple medical institutions and perform prospective studies to validate the generalization and accuracy of DLRPM. Secondly, All the features of radiomics are derived from the venous phase images of CECT and lack of diversity. In the future, multiphase CT data can be collected for feature enrichment.Finally,VOI segmentation of tumor is not automatic, and the probability of error in artificial semi-automatic segmentation is large and difficult to find. This may be overcome by automatic segmentation artificial intelligence system in the future.

In conclusion, we established DLRPM based on the characteristics of radiomics and pathomics to predict the complete pathological response of breast cancer patients to neoadjuvant chemotherapy. This model can help clinicians accurately predict the efficacy of neoadjuvant chemotherapy before treatment, highlighting the potential of artificial intelligence to improve the personalized treatment of breast cancer patients.

## Supplementary Information

**Additional file 1:** **Figure S1.** VOI segmentation. (A) The process of lesion delineation in a patient with breast cancer who achieved pCR in the evaluation of the efficacy of NAC. (B) The process of lesion delineation in a patient with breast cancer who achieved Non-pCR in the evaluation of the efficacy of NAC. **Figure S2.** (A)Segmentation Process of WSI, (B)color normalization. **Figure S3.** Image preprocessing in CellProBased on the “Unmix Colors” module to separate H&E-stained images and convert them into haematoxylin-stained and eosin-stained greyscale images, The H&E-stained images were also converted to greyscale images using the “ColorToGray” module. **Figure S4.** (A)Pipeline 1. First, greyscale H&E, haematoxylin and eosin images were assessed by using the “MeasureImageQuality” module with three types of features, including blur features, intensity features and threshold features. Subsequently, ‘MeasureColocalization’ module measured the colocalization and correlation between intensities in haematoxylin images and eosin images on a pixel-by-pixel basis. Next, ‘MeasureGranularity’ module outputed spectra of size measurements of the textures in three types of images. Finally, ‘MeasureTexture’ module measured the degree and nature of textures within three types of images to quantify their roughness and smoothness. (B)Pipeline 2. Haematoxylin-stained images were segmented via ‘IdentifyPrimaryObjects’module and‘IdentifySecondaryObjects’ module ,Quantitative image features of object shape, size, texture, and pixel intensity distribution were further extracted via multiple modules, including measure models of ‘Object Intensity Distribution’, ‘Object Intensity’, ‘Texture’, and ‘Object Size Shape’. **Table S1.** Baseline characteristics in the training and validation Sets. **Table S2.** The selected radiomics features. **Table S3.** The selected pathomics features. **Table S4.** The selected deep learning pathomics features.

## Data Availability

The datasets used and/or analyzed during the current study are available from the corresponding author on reasonable request.
