# Supplementary material for: Development and validation of a radiopathomic model for predicting pathologic complete response to neoadjuvant chemotherapy in breast cancer patients
Source: BMC Cancer. 2023 May 12;23:431. doi: 10.1186/s12885-023-10817-2 (PMC10176880; doi:10.1186/s12885-023-10817-2)
Supplement: Supplementary file 1 — Additional file 1: Figure S1. VOI segmentation. (A) The process of lesion delineation in a patient with breast cancer who achieved pCR in the evaluation of the efficacy of NAC. (B) The process of lesion delineation in a patient with breast cancer who achieved Non-pCR in the evaluation of the efficacy of NAC. Figure S2. (A)Segmentation Process of WSI, (B)color normalization. Figure S3. Image preprocessing in CellProBased on the “Unmix Colors” module to separate H&E-stained images and convert them into haematoxylin-stained and eosin-stained greyscale images, The H&E-stained images were also converted to greyscale images using the “ColorToGray” module. Figure S4. (A)Pipeline 1. First, greyscale H&E, haematoxylin and eosin images were assessed by using the “MeasureImageQuality” module with three types of features, including blur features, intensity features and threshold features. Subsequently, ‘MeasureColocalization’ module measured the colocalization and correlation between intensities in haematoxylin images and eosin images on a pixel-by-pixel basis. Next, ‘MeasureGranularity’ module outputed spectra of size measurements of the textures in three types of images. Finally, ‘MeasureTexture’ module measured the degree and nature of textures within three types of images to quantify their roughness and smoothness. (B)Pipeline 2. Haematoxylin-stained images were segmented via ‘IdentifyPrimaryObjects’module and‘IdentifySecondaryObjects’ module ,Quantitative image features of object shape, size, texture, and pixel intensity distribution were further extracted via multiple modules, including measure models of ‘Object Intensity Distribution’, ‘Object Intensity’, ‘Texture’, and ‘Object Size Shape’. Table S1. Baseline characteristics in the training and validation Sets. Table S2. The selected radiomics features. Table S3. The selected pathomics features. Table S4. The selected deep learning pathomics features. [file 12885_2023_10817_MOESM1_ESM.docx]

**Figure S1**


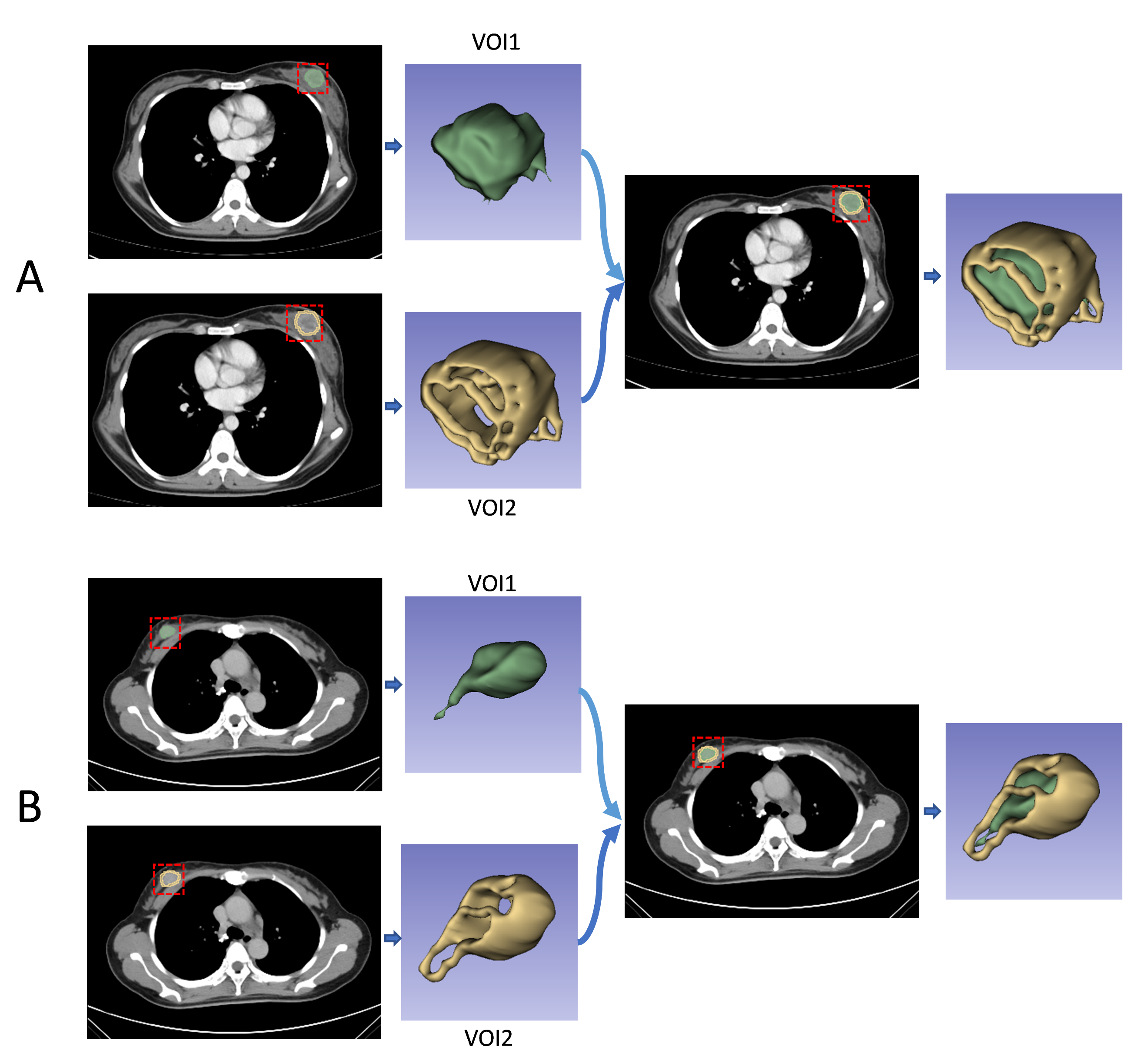


Figure S1. **VOI segmentation.** (A) The process of lesion delineation in a patient with breast cancer who achieved pCR in the evaluation of the efficacy of NAC. (B) The process of lesion delineation in a patient with breast cancer who achieved Non-pCR in the evaluation of the efficacy of NAC.

**Figure S2**

Figure S2. (A)Segmentation Process of WSI, (B)color normalization.

**Figure S3**


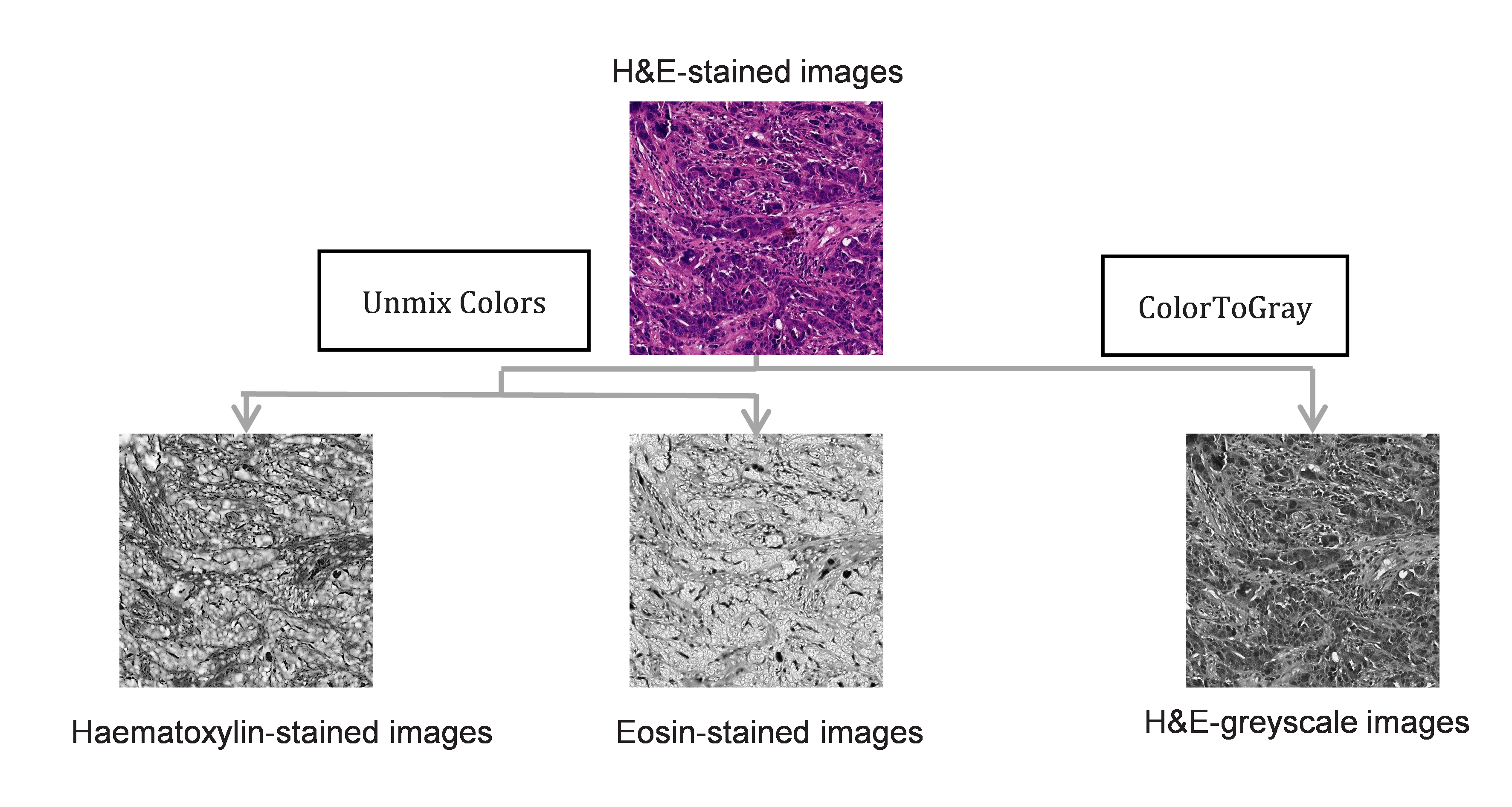


Figure S3. Image preprocessing in CellProBased on the “Unmix Colors” module to separate H&E-stained images and convert them into haematoxylin-stained and eosin-stained greyscale images, The H&E-stained images were also converted to greyscale images using the “ColorToGray” module.

**Figure S4**


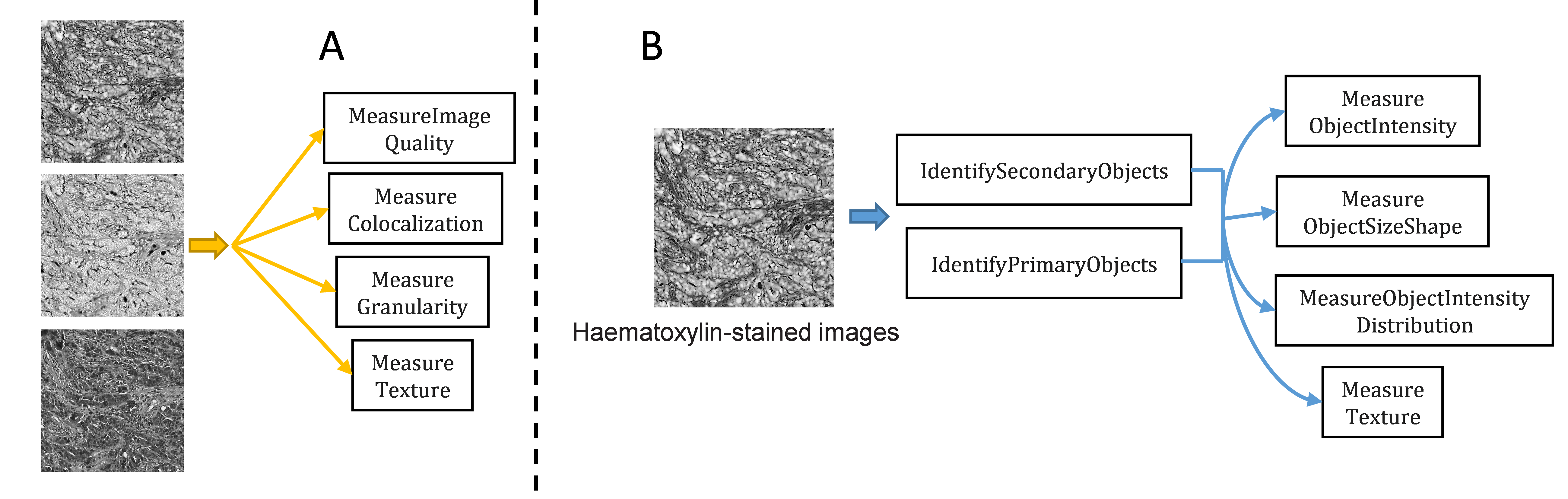


Figure S4. (A)Pipeline 1. First, greyscale H&E, haematoxylin and eosin images were assessed by using the “MeasureImageQuality” module with three types of features, including blur features, intensity features and threshold features. Subsequently, ‘MeasureColocalization’ module measured the colocalization and correlation between intensities in haematoxylin images and eosin images on a pixel-by-pixel basis. Next, ‘MeasureGranularity’ module outputed spectra of size measurements of the textures in three types of images. Finally, ‘MeasureTexture’ module measured the degree and nature of textures within three types of images to quantify their roughness and smoothness. (B)Pipeline 2. Haematoxylin-stained images were segmented via ‘IdentifyPrimaryObjects’module and‘IdentifySecondaryObjects’ module ,Quantitative image features of object shape, size, texture, and pixel intensity distribution were further extracted via multiple modules, including measure models of ‘Object Intensity Distribution’, ‘Object Intensity’, ‘Texture’, and ‘Object Size Shape’.

**Table S1** Baseline characteristics in the training and validation Sets.

| Characteristics | Training set(n=155) | Validation set(n=56) | *P* |
| --- | --- | --- | --- |
| Age | 49.0(46.0,55.0) | 49.5(45.0,56.2) | 0.627 |
| T stage (%) |  |  | 0.925 |
| T1 | 7 (4.52) | 3 (5.36) |  |
| T2 | 111 (71.6) | 41 (73.2) |  |
| T3 | 18 (11.6) | 7 (12.5) |  |
| T4 | 19 (12.3) | 5 (8.93) |  |
| N stage (%) |  |  | 0.110 |
| N0 | 41 (26.5) | 14 (25.0) |  |
| N1 | 94 (60.6) | 29 (51.8) |  |
| N2 | 17 (11.0) | 8 (14.3) |  |
| N3 | 3 (1.94) | 5 (8.93) |  |
| ER status (%) |  |  | 0.859 |
| Negative | 76 (49.0) | 26 (46.4) |  |
| Positive | 79 (51.0) | 30 (53.6) |  |
| PR status (%) |  |  | 0.280 |
| Negative | 60 (38.7) | 27 (48.2) |  |
| Positive | 95 (61.3) | 29 (51.8) |  |
| HER2 status (%) |  |  | 0.611 |
| Negative | 91 (58.7) | 30 (53.6) |  |
| Positive | 64 (41.3) | 26 (46.4) |  |
| Ki-67 status (%) |  |  | 1.000 |
| <30% | 37 (23.9) | 13 (23.2) |  |
| ≥30% | 118 (76.1) | 43 (76.8) |  |

Note:Data in parentheses are percentages; p values were derived from the univariate analysis between each of characteristic and pCR Status.

**Table S2** The selected radiomics features.

| Radiomics Features(n=30) |
| --- |
| exponential_glszm_SizeZoneNonUniformityNormalized |
| exponential_glszm_SmallAreaLowGrayLevelEmphasis |
| lbp-3D-k_firstorder_Skewness |
| lbp-3D-k_glszm_SizeZoneNonUniformityNormalized |
| lbp-3D-m1_gldm_DependenceVariance |
| lbp-3D-m2_glcm_ClusterShade |
| lbp-3D-m2_glcm_InverseVariance |
| log-sigma-4-0-mm-3D_glszm_SmallAreaLowGrayLevelEmphasis |
| log-sigma-5-0-mm-3D_firstorder_Skewness |
| logarithm_gldm_SmallDependenceLowGrayLevelEmphasis |
| logarithm_glrlm_RunVariance |
| squareroot_glcm_Imc2 |
| wavelet-HLH_glcm_Correlation |
| wavelet-HLH_glszm_SmallAreaEmphasis |
| wavelet-HLL_firstorder_Skewness |
| wavelet-LLH_firstorder_10Percentile |
| PR_exponential_glszm_ZonePercentage |
| PR_gradient_glcm_Correlation |
| PR_lbp-3D-k_glszm_SmallAreaHighGrayLevelEmphasis |
| PR_log-sigma-2-0-mm-3D_glcm_InverseVariance |
| PR_log-sigma-5-0-mm-3D_glcm_DifferenceEntropy |
| PR_wavelet-HLH_glcm_DifferenceEntropy |
| PR_wavelet-HLL_firstorder_InterquartileRange |
| PR_wavelet-HLL_firstorder_Skewness |
| PR_wavelet-HLL_glszm_ZoneVariance |
| PR_wavelet-LHL_firstorder_RootMeanSquared |
| PR_wavelet-LHL_glcm_ClusterShade |
| PR_wavelet-LLH_firstorder_Skewness |
| PR_wavelet-LLH_glszm_ZonePercentage |
| PR_wavelet-LLL_firstorder_10Percentile |

Note: PR= peritumoral region.

**Table S3** The selected pathomics features.

| Pathomics Features(n=12) |
| --- |
| ExecutionTime_09MeasureGranularity |
| Granularity_15_Hematoxylin |
| Granularity_5_OrigGray |
| Granularity_6_Eosin |
| Granularity_8_Eosin |
| Granularity_9_Eosin |
| H_Mean_IdentifySecondaryObjects_AreaShape_Zernike_8_2 |
| H_Median_IdentifySecondaryObjects_AreaShape_Zernike_4_4 |
| H_StDev_IdentifyPrimaryObjects_AreaShape_Zernike_9_9 |
| H_StDev_IdentifySecondaryObjects_AreaShape_Zernike_6_4 |
| H_StDev_IdentifySecondaryObjects_AreaShape_Zernike_7_3 |
| H_StDev_IdentifySecondaryObjects_Texture_Correlation_Hematoxylin_3_03_256 |
| **Table S4** The selected deep learning pathomics features. |
| Deep Learining Pathomics Features(n=8) |
| DLP_0 |
| DLP_2 |
| DLP_3 |
| DLP_5 |
| DLP_51 |
| DLP_61 |
| DLP_62 |
| DLP_80 |
